# Mixed reality-guided intraoperative visual support for combined intracavitary and interstitial brachytherapy for cervical cancer^☆^

**DOI:** 10.1016/j.tipsro.2026.100408

**Published:** 2026-04-26

**Authors:** Ryuta Hirai, Tomohiro Ohta, Mitsunobu Igari, Yu Kumazaki, Jun Watanabe, Keita Tsukahara, Misaki Iino, Yasuhiro Ryuno, Tomomi Aoshika, Satoshi Saito, Takanori Abe, Shin-ei Noda, Shingo Kato

**Affiliations:** Department of Radiation Oncology, Saitama Medical University International Medical Centre, 1397-1 Yamane, Hidaka, Saitama 350-1298, Japan

**Keywords:** Uterine cervical cancer, Brachytherapy, Intraoperative, Mixed reality, Virtual reality, Head-mounted display

## Abstract

**Purpose:**

This study aimed to develop and evaluate a mixed reality-guided intraoperative visual support system for safe and accurate needle applicator insertion for combined intracavitary and interstitial brachytherapy (IC/IS-BT).

**Materials and Methods:**

A QR code was printed as a position and scale alignment reference when merging the real and virtual worlds. The QR code was positioned near the RANDO phantom, and MRI and CT scanning were performed. Treatment planning data, including the target and organs at risk structures and dose distribution from a cervical cancer patient who underwent brachytherapy, were attached to MRI and CT images of the RANDO phantom using a radiation treatment planning system and stored as DICOM data. DICOM data were then converted to object files (OBJ) that represent 3D shapes. These OBJ files were imported into the computer-generated virtual world, and the mixed reality application was built and executed on head-mounted displays (HMDs).

**Results:**

The system successfully merged the real and virtual worlds through the HMD, displaying 3D support data on the phantom. This system allows all HMD wearers to share 3D support data. Positions and scales between the real and virtual worlds are aligned using the upper left-hand corner of the QR code as the reference point, and the worlds are merged. Phantom-based evaluations demonstrated that projection errors measured by Operator A were 3.1 ± 1.1 mm in the anterior–posterior (A–P) direction and 2.6 ± 1.1 mm in the right–left (R–L) direction; corresponding projection errors for Operator B were 2.1 ± 1.4 mm in the A–P direction and 2.4 ± 1.1 mm in the R–L direction (n = 10 measurements per operator). The average frame rate was 69.7 ± 11.5 fps, corresponding to a latency of 14 msec.

**Conclusions:**

This report presents the system configuration, accuracy assessment and potential clinical applications for IC/IS-BT.

## Highlights

• We used DICOM RT data as mixed reality-based support data for brachytherapy in gynaecology.
• The real and virtual worlds were merged through head-mounted display, and 3D models were displayed.
• This system will lower the hurdles for interstitial needle applicator insertion.

## Introduction

Intracavitary brachytherapy (ICBT) has a key role in radiotherapy for uterine cervical cancer. With technological advancements over the past two decades, three-dimensional image-guided brachytherapy (3D-IGBT) has made it possible to deliver a high radiation dose to the cervical tumour while reducing the dose to adjacent organs at risk (OARs), such as the rectum and bladder, which contributes to good local control and improved survival [1], [2], [3], [4], [5], [6]. However, since the rectum and bladder tend to be near the primary cervical tumour, if patients have extensive and bulky tumours, it can be difficult to achieve dose constraints in ICBT.

Meanwhile, combined intracavitary and interstitial brachytherapy (IC/IS-BT) improves dose distributions by inserting interstitial needle applicators (needles) into areas of tumour infiltration, allowing the delivery of adequately high radiation doses to the tumour while minimising doses to the OARs. Many clinical studies have reported favorable local control outcomes with low rates of radiation toxicity [5], [7], [8], [9]. It has also been reported that an appropriate increase in the number of needles can improve dose distribution [10], [11], [12].

Needles are inserted intraoperatively by the operator while generally referring to ultrasound images [13]. In current clinical practice, all imaging used for applicator insertion and image-guided brachytherapy (IGBT) are presented either as axial, coronal and sagittal sectional images or pseudo-three-dimensional (3D) images displayed on external displays, which makes needle applicator insertion more challenging [10], [13], [14]. Moreover, the visual field available through direct observation using gynaecological instruments is limited. Furthermore, it is difficult to insert needles correctly into the uterus, as its position and shape change significantly from fraction to fraction, and there is a risk of accidental insertion into the adjacent rectum, colon and intestine [15]. Conventional 3D-IGBT remains an essential technique because it enables accurate positioning of the irradiation site based on imaging. However, it does not provide direct guidance for determining the insertion route of intracavitary or interstitial applicators. Furthermore, the challenges described above may make it difficult to insert needles at optimal locations.

Extended reality (XR), including virtual reality (VR) and mixed reality (MR), has made significant progress in recent years and is increasingly being applied in the medical field [16], [17], [18], [19]. The real world refers to the physical environment constructed from visual information perceived directly by the user through unaided vision. In contrast, the virtual world is an intangible information space generated by a computer and presented to the user’s field of view via displays. VR, the most widely known of these technologies, is fully immersive; we are in a different environment or world apart from the real world. Mixed reality is a technology that merges the real and virtual worlds to build a space in which they interact in real time [20]. Users can receive visual information in the virtual world through devices such as head-mounted display (HMD) while maintaining their field of view in the real world.

In addition to conventional 3D image-guidance, the proposed system employs mixed reality technology to project 3D-brachytherapy treatment planning data directly onto the patient’s body without compromising the information obtained through standard image-guided techniques. This approach enhances the operator’s understanding of the patient’s anatomical structures, potentially contributing to improved decision-making regarding needle applications. Building upon and extending the principles of conventional 3D-IGBT, we have termed this technique “mixed reality-guided brachytherapy”. In this technical report, we introduce the system configuration and its details.

## Materials and Methods

### Overview of mixed reality-based system development

**Fig. 1:**
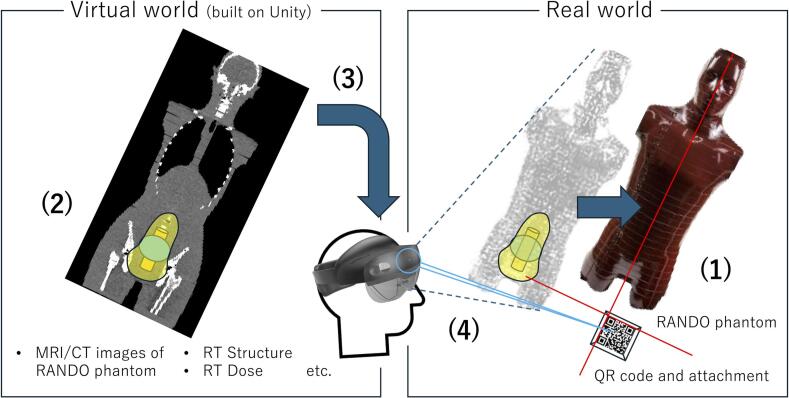
Schematic of the procedural flow of merging worlds. (1) The QR code is positioned on the MRI/CT couch such that the upper left-hand corner of the QR code is aligned with a patient alignment laser, and the CT centre is set. The RANDO phantom is positioned near the QR code, and MRI/CT scanning is performed. (2) Brachytherapy treatment planning is performed, and treatment plan data are stored as DICOM RT files. These DICOM data are converted to an object file (OBJ). (3) OBJ data are imported into the virtual world. (4) The user wears the head-mounted display and looks at the QR code. The positions and scales between the real and virtual worlds are aligned using the QR code, and these worlds are merged.

A conceptual diagram is shown in Fig. 1. HoloLens 2 (Microsoft Corporation, Redmond, WA, USA) was used as the head-mounted display (HMD). The system was developed using Unity (Unity Software Inc., San Francisco, CA, USA). Unity enables graphical user interface (GUI)-based application development by placing various objects (2D and three-dimensional (3D) models, videos, images, etc.) in a 2D or 3D computer-generated virtual world.

The Microsoft Mixed Reality Toolkit (MRTK) for Unity was used to enable spatial mapping, gesture recognition and QR code tracking. Visual Studio 2022 (Microsoft Corporation, Redmond, WA, USA) was used to build and deploy mixed reality applications that run on HoloLens 2. These tools were used to integrate 3D treatment planning data and develop the mixed reality application for visualization through the HMD.

### QR code system for intraoperative linkage between real and virtual worlds

A 10 cm × 10 cm QR code was generated and printed as a position and scale alignment reference when merging the real and virtual worlds [21]. The visibility of the QR code was improved by adding a 1-cm margin around the QR code. The attachment was produced using a 3D printer, and the QR code was fixed on this attachment. The attachment was made of carbon fiber reinforced polyamide 12 and was visible on CT images. The base of the attachment was designed to be able to contain water, and the attachment was also visible on MRI images.

The upper left-hand corner of the QR code was set as the reference point in the real world. To align the virtual world with the real world through the HMD, a C# script code in which the reference point in the virtual world tracks the upper left-hand corner of the QR code was created. When the QR code was recognised by the camera positioned in front of HoloLens 2, the virtual and real-world locations were automatically synchronised, linked and aligned. In this scene, the side length of the QR code was recognised by the HoloLens 2 camera and used to align the scale with the virtual world. The size of the projected data is expected to have at most a 1% error from the actual size [21]. For example, a 10 cm code can show a measurement error of ±1 mm. Although this estimate is based on Microsoft Learn, a non-peer-reviewed technical documentation source, the positional accuracy of the developed system was quantitatively evaluated in this study, as detailed in a later section.

### Creating 3D support models using radiation treatment planning DICOM data

An anthropomorphic RANDO phantom (Alderson Research Laboratories, Stanford, CT) was used in this study (Fig. 1). For treatment planning CT imaging, the QR code and attachment were positioned on the CT couch such that the upper left-hand corner of the QR code was aligned with a patient alignment laser, and the CT centre was set. As a result of this alignment, the upper-left corner of the QR code (defined as the reference point) was assigned the coordinate (x, y, z) = (0, 0, 0) in the CT images. At our institution, sub-millimetre alignment between the CT laser and the CT image centre is ensured through daily and monthly QA procedures. The RANDO phantom was positioned near the QR code, and CT scanning was performed. The “Real world” illustration in Fig. 1 depicts this setup. As with CT imaging, the RANDO phantom was set up to acquire MRI images.

In clinical practice, organ contours and dose distribution are created using a radiation treatment planning system (RTPS) based on the patient’s MRI/CT images. However, since the RANDO phantom does not contain any internal organs or any space to insert intracavitary applicators, it is not possible to generate contours and dose distributions using the standard clinical workflow. To address this limitation, anonymised DICOM radiation treatment (RT) structures (uterus and target, rectum and bladder) and RT dose of a patient who underwent ICBT in actual clinical practice were attached to MRI and CT images of the RANDO phantom using a RTPS. The patient dataset was selected to approximate the body habitus of the phantom. The transverse pelvic width of the RANDO phantom was 37 cm, and a patient dataset with a transverse pelvic width of 34 cm and a body mass index (BMI) of 20.9 was selected to represent a standard body habitus.

To implement the system shown in Fig. 1, DICOM data associated with MRI/CT images for treatment planning were converted into OBJ files representing three-dimensional structures. OBJ is a data format that represents 3D shapes as a collection of small polygons and was used as a 3D model for visual support. These OBJ files were imported into the Unity development environment, where they were placed in a computer-generated virtual space while maintaining the original coordinate system. Unity generated a solution file (SLN), which was opened in Visual Studio 2022 to build the mixed reality application for intraoperative visual support. The application was then deployed to the HoloLens 2 device via Wi-Fi and ran. Through this process, the developed system enabled the real and virtual environments to be merged and allowed the 3D support models to be displayed on the RANDO phantom using the upper-left corner of the QR code as the reference point.

### Measurement of positional accuracy and latency of projected 3D support models

**Fig. 2:**
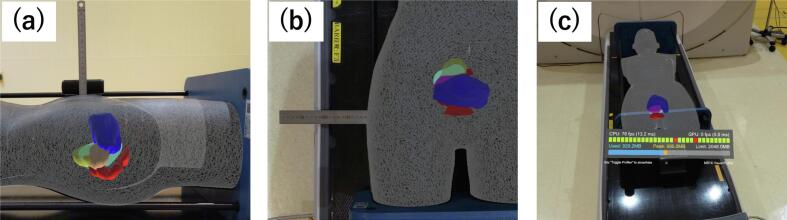
Measurement of positional accuracy and latency.

A ruler was affixed to the abdominal surface of the RANDO phantom placed in the real world, and the 3D models were projected on the RANDO phantom. The positional error between the RANDO phantom and the 3D models of the body surface generated by surface rendering of the CT images imported into the virtual space was measured in millimetres to acquire captured images (anterior–posterior direction) (A–P) (Fig. 2(a)). Additionally, a ruler was placed beneath the RANDO phantom, and the positional error was measured in the right–left (R–L) direction by observing and capturing the phantom from a frontal view (Fig. 2(b)). To assess reproducibility, the user’s gaze was intentionally diverted from the phantom and then returned, and the display was consistency re-evaluated. Each measurement (A–P and R–L) was repeated 10 times. Measurements were independently performed by two operators. Differences in positional errors between operators were evaluated using the Mann–Whitney *U* test, with statistical significance defined as p < 0.05.

We also implemented a function within the system to continuously monitor the frame rate to evaluate the latency of the system (Fig. 2(c)). As with the investigation mentioned above, the user’s gaze was intentionally diverted from the phantom and then returned, and the display re-cast time was evaluated. This measurement was also repeated 10 times.

### Intended clinical workflow

In clinical settings, MRI or CT imaging would be performed after intracavitary applicator insertion. Treatment planning DICOM data would then be converted to OBJ files and projected as 3D support models intraoperatively to support interstitial needle placement. The patient does not necessarily need to remain on the CT/MRI table. However, positional displacement between the patient and the QR code must be minimized. If the patient is moved after imaging, changes in body posture (for example, changes in leg angle) should be minimized to maintain spatial consistency between the real and virtual environments. In cases where patient posture changes, surface-rendered body structures may serve as visual landmarks to assist with realignment. This concept is consistent with previously reported techniques for patient setup in external beam radiotherapy [22], [23].

## Results

**Fig. 3:**
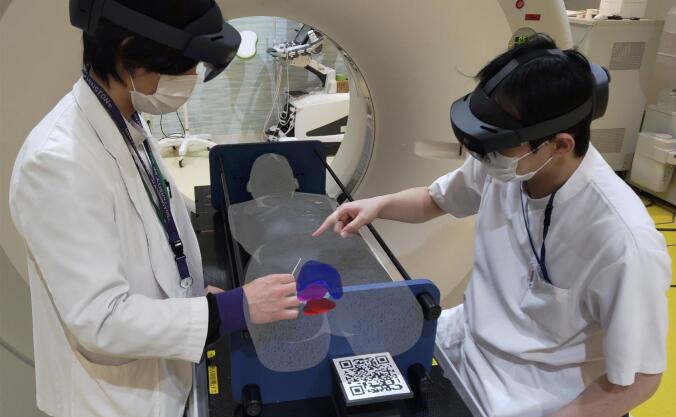
Projection of mixed reality-based 3D support data. A QR code was placed near the RANDO phantom. CT images of the RANDO phantom were surface rendered and displayed as white meshes, and the structures of the target, rectum and bladder were displayed as magenta, red or blue solid. The photograph shows the state after CT imaging has been completed, with the patient and treatment table moved out of the CT gantry bore. (For interpretation of the references to colour in this figure legend, the reader is referred to the web version of this article.)

OBJ data converted from DICOM data were automatically placed in the computer-generated virtual world while maintaining the coordinate system. The developed system merged the real and virtual worlds through the HMD. The 3D support models were displayed on the RANDO phantom using the upper left-hand corner of the QR code as the reference point (Fig. 3). The operators wore the HMD on their heads and looked at the QR code, and the camera at the centre of the HMD automatically recognised the QR code. The positions and scales between the real and virtual worlds were aligned using the QR code, and these worlds were merged.

**Fig. 4:**
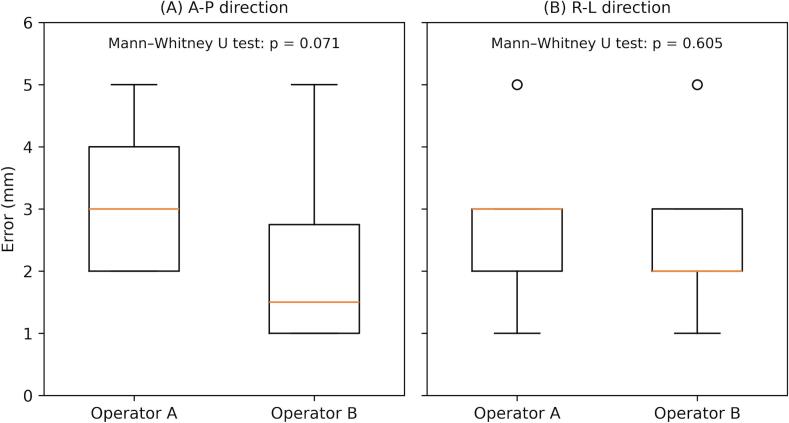
Box plots of projection position errors measured by two operators in the anterior–posterior (A–P) and right–left (R–L) directions.

The resulting errors of the RANDO phantom and 3D models of the body surface were evaluated by two independent operators. Operator A measured projection errors of 3.1 ± 1.1 mm in the A–P direction and 2.6 ± 1.1 mm in the R–L direction. Operator B measured projection errors of 2.1 ± 1.4 mm in the A–P direction and 2.4 ± 1.1 mm in the R–L direction. Fig. 4 illustrates the distribution of positional errors for each operator. Although a trend toward smaller errors for Operator B was observed in the A–P direction, no statistically significant inter-operator differences were noted in either the A–P or R–L directions.

This system allowed all HMD wearers to share 3D support data. According to the movement of the HMD wearer, the 3D models were displayed in a coordinated position with the RANDO phantom without any time lag. We conducted 10 capture sessions and measured an average frame rate of 69.7 frames per second (fps), which corresponds to an average time lag of approximately 14 ms.

In this study, DICOM RT structures (target, rectum and bladder) and dose distribution (6 Gy isodose area) obtained from RTPS and surface rendered CT images of the RANDO phantom were displayed as solid, translucent, and mesh in 3D support models. The user experience can be enhanced by changing the display patterns (solid/translucent, wires, meshes, dots, etc.) and the combination of projected support data. Using these 3D models, it was also possible for the operator and several staff members to share support data while discussing and assessing the needle insertion depth and angle (Fig. 3).

## Discussion

In recent years, active research using XR has been reported in the field of radiation therapy. For example, XR has been used in clinical patient positioning for external beam radiotherapy (EBRT), as well as in non-clinical education using 360-degree videos depicting a first-person patient perspective during the EBRT care path to reduce patient anxiety, training for workflow, current experience of EBRT and treatment simulation for students, staff members and patients [22], [23], [24], [25], [26], [27], [28]. Moreover, in brachytherapy for cervical cancer, some have described the use of XR for simulation and training purposes [29], [30]. However, to the best of our knowledge, no study has reported on the use of DICOM RT data obtained from radiation treatment planning for XR-based intra-operative support for brachytherapy of gynaecological cancer.

We developed a mixed reality-based intraoperative visual support system that uses DICOM RT data imported into a virtual world as 3D support models and merged with the real world. We evaluated the positional accuracy and latency of the system and demonstrated that it operated stably and properly using an anthropomorphic phantom. The combination of mixed reality and DICOM is useful and versatile, as DICOM offers excellent data integrity, readability and preservability, and fulfils the essential requirements of mixed reality to ensure the quality of supporting data.

One potential advantage of mixed reality-guided brachytherapy is the ability to visually identify anatomical structures such as the rectum, bladder and small bowel, which should be avoided during needle insertion. This visualization may support the selection of safe needle trajectories and, consequently, effectively guide radiation dose delivery. We believe that these features will lower the hurdles to the introduction and implementation of ICIS-BT and increase its usage. Moreover, it is also possible to discuss and assess needle insertion in pretreatment planning and post-operative evaluation. Our final goal is to improve outcomes (i.e., improved tumour control and reduced incidence of failure) for bulky cervical cancer through safe and accurate needle insertion.

Our system may also be applicable in non-clinical settings, as described in previous reports on EBRT and brachytherapy [24], [25], [26], [27], [28], [29], [30], such as teaching and mentoring for residents and students by senior doctors, and sharing information with other professionals or patients. By providing enough HMDs, several doctors, medical staff, students and patients can visually share 3D support models. This system, which uses DICOM RT data, can provide effective and efficient education and guidance by sharing abundant information, and may contribute to resolving the shortage of human resources, with possible use for teaching from remote locations.

This study has some limitations. First, the system generates support data based on radiation treatment planning DICOM data created for MRI/CT images, and therefore does not provide real-time information. In particular, the insertion of needles may immediately alter the position of individual organs. Second, it should be noted that the current system provides only indirect guidance for implantation of needles. Direct guidance of the needle insertion point and trajectory has yet to be achieved and remains a future development goal. However, these limitations may be overcome by supplementing real-time data through the combined use of ultrasound images, as employed in conventional 3D IGBT. Third, the positional relationship between the patient and the QR code is very important, and the positional accuracy of the support data is dominated by the accuracy of patient fixation. To achieve more stable alignment and scaling of 3D support models, improvements in QR code recognition performance may be required through technical ingenuity [22], [23], [31]. Currently, the creation of 3D visual support models also requires a data conversion step from DICOM to OBJ.

However, even considering these limitations, we believe this system will provide surgeons with a large amount of information intraoperatively, which will be of great benefit. Importantly, the additional information provided through mixed reality does not interfere with, or diminish, the utility of conventional IGBT; rather, it serves as a complementary tool to enhance the procedure.

In this report, we introduced the system configuration of a mixed reality-based intraoperative visual support system and its accuracy as well as potential clinical applications for IC/IS-BT. This approach may improve intraoperative decision-making and enhance the safety and feasibility of brachytherapy for cervical cancer. We are aiming for its practical application through collaborations with the industry and academia.

## Grant support

This research was partially supported by JSPS KAKENHI (Grant Number 22K15829 and 25K19149) to RH.

## Declaration of competing interest

The authors declare the following financial interests/personal relationships which may be considered as potential competing interests: Ryuta Hirai holds patent JP7653730 licensed to Saitama Medical University and patent PCT/JP2023/003666 pending, which are related to the technological field of this study. If there are other authors, they declare that they have no known competing financial interests or personal relationships that could have appeared to influence the work reported in this paper. Ryuta Hirai, Tomohiro Ohta, Mitsunobu Igari, Yu Kumazaki, Jun Watanabe, Keita Tsukahara, Misaki Iino, Yasuhiro Ryuno, Tomomi Aoshika, Satoshi Saito, Takanori Abe, Shin-ei Noda, Shingo Kato.

## Data Availability

The data that support the findings of this study are available from the corresponding author, RH, upon reasonable request.
